# Bis[2-(2-oxoindolin-3-yl­idene)-*N*-phenylhydrazinecarbothio­amidato-κ^3^ *O*,*N* ^2^,*S*]nickel(II) dimethyl­formamide monosolvate

**DOI:** 10.1107/S1600536812012834

**Published:** 2012-04-04

**Authors:** Amna Qasem Ali, Naser Eltaher Eltayeb, Siang Guan Teoh, Abdussalam Salhin, Hoong-Kun Fun

**Affiliations:** a School of Chemical Sciences, Universiti Sains Malaysia, Minden, Penang, Malaysia; b Faculty of Science, Sabha University, Libya; c Department of Chemistry, International University of Africa, Khartoum, Sudan; d X-ray Crystallography Unit, School of Physics, Universiti Sains Malaysia, 11800 USM, Penang, Malaysia

## Abstract

The asymmetric unit of the title compound, [Ni(C_15_H_11_N_4_OS)_2_]·C_3_H_7_NO, contains one Ni^II^ complex mol­ecule and one disordered dimethyl­formamide solvent mol­ecule. The Ni^II^ ion is six-coordinated in a distorted octa­hedral geometry by two N, two O and two S atoms. An intra­molecular C—H⋯S hydrogen bond generates an *S*(6) ring motif. In the crystal, mol­ecules are linked through inter­molecular N—H⋯S, N—H⋯O, C—H⋯N, C—H⋯O and C—H⋯S hydrogen bonds into infinite two-dimensional network parallel to the *ab* plane. The structure is further stablized by weak C—H⋯π inter­actions. The dimethylformamide solvent molecule is disordered over two sets of sites in a 0.514 (15):0.486 (15) ratio.

## Related literature

For related structures, see: Qasem Ali *et al.* (2011*a* ▶,*b* ▶, 2012*a* ▶,*b* ▶); Ali *et al.* (2012 ▶). For the biological activity of Schiff bases, see: Bhandari *et al.* (2008 ▶); Bhardwaj *et al.* (2010 ▶); Pandeya *et al.* (1999 ▶); Sridhar *et al.* (2002 ▶); Suryavanshi & Pai (2006 ▶). For the cytotoxic and anti­cancer activity of isatin and its derivatives, see: Vine *et al.* (2009 ▶). For graph-set analysis, see: Bernstein *et al.* (1995 ▶). For bond-length data, see: Allen *et al.* (1987 ▶).
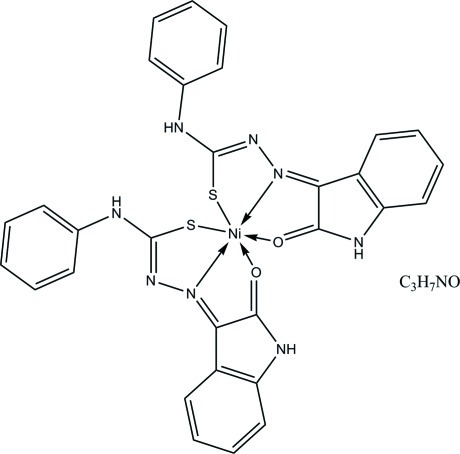


## Experimental

### 

#### Crystal data

[Ni(C_15_H_11_N_4_OS)_2_]·C_3_H_7_NO
*M* *_r_* = 722.48
Triclinic, 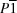

*a* = 12.2491 (2) Å
*b* = 12.3170 (3) Å
*c* = 13.1142 (2) Å
α = 104.854 (1)°
β = 112.943 (1)°
γ = 102.798 (1)°
*V* = 1642.31 (5) Å^3^
*Z* = 2
Mo *K*α radiation
μ = 0.77 mm^−1^
*T* = 100 K
0.45 × 0.15 × 0.13 mm

#### Data collection

Bruker APEXII CCD diffractometer
Absorption correction: multi-scan (*SADABS*; Bruker, 2005 ▶) *T*_min_ = 0.723, *T*_max_ = 0.906
34412 measured reflections
9435 independent reflections
7397 reflections with *I* > 2σ(*I*)
*R*_int_ = 0.033

#### Refinement

*R*[*F* ^2^ > 2σ(*F* ^2^)] = 0.041
*wR*(*F* ^2^) = 0.107
*S* = 1.03
9435 reflections
481 parameters
25 restraints
H atoms treated by a mixture of independent and constrained refinement
Δρ_max_ = 0.63 e Å^−3^
Δρ_min_ = −0.37 e Å^−3^

### 

Data collection: *APEX2* (Bruker, 2005 ▶); cell refinement: *SAINT* (Bruker, 2005 ▶); data reduction: *SAINT*; program(s) used to solve structure: *SHELXS97* (Sheldrick, 2008 ▶); program(s) used to refine structure: *SHELXL97* (Sheldrick, 2008 ▶); molecular graphics: *SHELXTL* (Sheldrick, 2008 ▶); software used to prepare material for publication: *SHELXTL* and *PLATON* (Spek, 2009 ▶).

## Supplementary Material

Crystal structure: contains datablock(s) I, global. DOI: 10.1107/S1600536812012834/zj2062sup1.cif

Structure factors: contains datablock(s) I. DOI: 10.1107/S1600536812012834/zj2062Isup2.hkl

Additional supplementary materials:  crystallographic information; 3D view; checkCIF report

## Figures and Tables

**Table 1:** Selected bond lengths (Å)

Ni1—N2	2.0342 (15)
Ni1—N6	2.0373 (15)
Ni1—O1	2.1886 (13)
Ni1—O2	2.2441 (13)
Ni1—S1	2.3564 (5)
Ni1—S2	2.3866 (5)

**Table 2:** Hydrogen-bond geometry (Å, °) *Cg*5, *Cg*7, *Cg*9 and *Cg*10 are the centroids of the N1/C1/C8/C7/C2, C2–C7, C17–C22 and C25–C30 rings, respectively.

*D*—H⋯*A*	*D*—H	H⋯*A*	*D*⋯*A*	*D*—H⋯*A*
N8—H1*N*8⋯S2^i^	0.86 (2)	2.48 (3)	3.301 (2)	159 (2)
N1—H1*N*1⋯O1^ii^	0.79 (4)	2.04 (4)	2.811 (2)	165 (4)
N5—H1*N*5⋯O3*X*^iii^	0.89 (4)	1.84 (4)	2.729 (9)	173 (3)
N4—H1*N*4⋯O2^iv^	0.82 (3)	2.22 (3)	3.006 (3)	161 (2)
C3—H3*A*⋯N7^ii^	0.95	2.56	3.500 (3)	173
C11—H11*A*⋯O2^iv^	0.95	2.55	3.349 (3)	142
C15—H15*A*⋯S1	0.95	2.53	3.194 (3)	127
C20—H20*A*⋯S1^v^	0.95	2.71	3.443 (2)	135
C30—H30*A*⋯S2^i^	0.95	2.84	3.551 (2)	132
C31*X*—H31*E*⋯*Cg*9^vi^	0.98	2.97	3.567 (11)	121
C31*X*—H31*F*⋯*Cg*7^iv^	0.98	2.79	3.460 (10)	126
C32*X*—H32*D*⋯*Cg*10^vii^	0.98	2.91	3.769 (12)	147
C31—H31*B*⋯*Cg*9^vi^	0.98	2.98	3.66 (2)	128
C31—H31*C*⋯*Cg*7^iv^	0.98	2.78	3.47 (2)	127
C32—H32*B*⋯*Cg*5^iv^	0.98	2.82	3.577 (16)	135

Symmetry codes: (i) 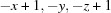; (ii) 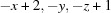; (iii) 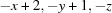; (iv) 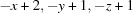; (v) 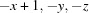; (vi) 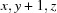; (vii) 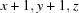.

**Fig. 1.**
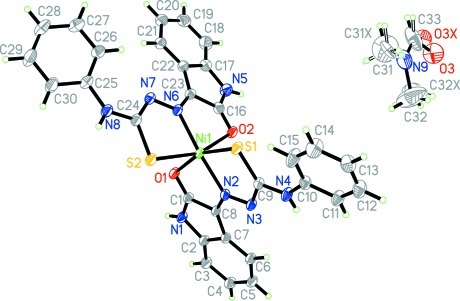
The molecular structure of the title compound, with 50% probability displacement ellipsoids and the atom-numbering scheme.

**Fig. 2.**
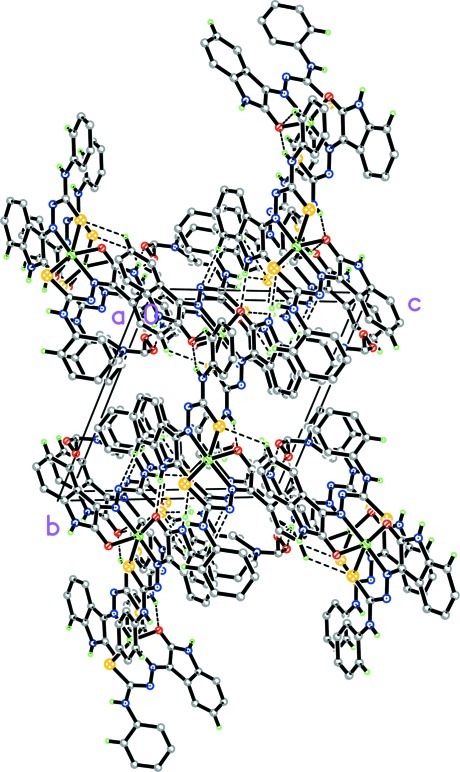
The crystal packing of the title compound viewed down the a axis. Hydrogen bonds are shown as dashed lines.

## supplementary crystallographic information

### Comment

Isatin (2,3-dioxindole) is an endogenous compound identified in humans, and its effect has been studied in a variety of systems. Biological properties of isatin and its derivatives include a range of actions in the brain, offer protection against bacterial (Suryavanshi & Pai, 2006) and fungal infections and possess anticonvulsant, anti-HIV (Pandeya *et al.*, 1999), anti-depressant and anti-inflammatory activities (Bhandari *et al.*, 2008). Recently, we reported the crystal structure of (*Z*)-*N*-methyl-2-(5-nitro-2-oxoindolin-3-ylidene) hydrazinecarbothioamide (Qasem Ali *et al.*, 2012*a*). In the present paper we describe the single-crystal X-ray diffraction study of title compound, Fig.1.

In the title compound, (Fig. 1), the asymmetric unit contains one Ni^II^ complex, [Ni(C_15_H_11_N_4_OS)_2_] and one solvent molecule, [C_3_H_7_NO], which displays disorder. Ni^II^ ion is six-coordinated in a distorted octahedral geometry by two N, two O and two S atoms. The Ni—N, Ni—O and Ni—S bond distances (Table 1) and the bond angles around Ni1 are normal (Allen *et al.*, 1987).

Intramolecular C15—H15A···S1 hydrogen bond generate an S(6) rings motif (Bernstein *et al.*, 1995)] (Table 2). Intramolecular interactions C26—H26A···N7 and C32X—H32D···O3X are also present.

In the crystal, molecules are linked through intermolecular N8—H1N8···S2, N1—H1N1···O1, N5—H1N5···O3X, N4—H1N4···O2, C3—H3A···N7, C11—H11A···O2, C20—H20A···S1 and C30—H30A···S2 hydrogen bonds into infinite two-dimensional network, (Table 1, Fig.2). Weak C—H···π interactions are also present: C31X—H31E···*Cg*9, C31X—H31F···*Cg*7, C32X—H32D···*Cg*10, C31—H31B···*Cg*9, C31—H31C···*Cg*7 and C32—H32B···*Cg*5 (Table 2). *Cg*5, *Cg*7, *Cg*9 and *Cg*10 are centroid of N1/C1/C8/C7/C2, C2—C7, C17—C22 and C25—C30 ring, respectively.

### Experimental

The Ni-complex has been synthesized by refluxing the reaction mixture of hot ethanolic solution (30 ml) of NiCl_2_ (0.01 mol) and hot ethanolic solution (30 ml) of the ligand as reported before (Qasem Ali, 2012*b*) (0.02 mol) for 2 hrs. The precipitate formed during reflux was filtered, washed with cold EtOH and recrystallized from hot EtOH. Yield (m.p.): 67% (>573 K). The green crystals were grown in acetone-DMF (4:1) by slow evaporation at room temperature.

### Refinement

N bound atoms were located in a difference Fourier map and were refined freely. The H atoms were positioned geometrically and refined using a riding model with C–H = 0.95 Å for aromatic ring and and C–H = 0.96–0.98 Å for methyl group with *U*_iso_(H) = 1.2*U*_eq_(C) and *U*_iso_(H) = 1.5*U*_eq_(C) for aromatic ring and methyl group respectively. The highest residual electron density peak is located at 0.97 Å from H33B and the deepest hole is located at 0.58 Å from N9.

### Crystal data

**Table:**

[Ni(C_15_H_11_N_4_OS)_2_]·C_3_H_7_NO	*Z* = 2
*M**_r_* = 722.48	*F*(000) = 748
Triclinic, *P*1	*D*_x_ = 1.461 Mg m^−^^3^
Hall symbol: -P 1	Mo *K*α radiation, λ = 0.71073 Å
*a* = 12.2491 (2) Å	Cell parameters from 9906 reflections
*b* = 12.3170 (3) Å	θ = 2.7–29.9°
*c* = 13.1142 (2) Å	µ = 0.77 mm^−^^1^
α = 104.854 (1)°	*T* = 100 K
β = 112.943 (1)°	Needle, green
γ = 102.798 (1)°	0.45 × 0.15 × 0.13 mm
*V* = 1642.31 (5) Å^3^	

### Data collection

**Table:**

Bruker APEXII CCD diffractometer	9435 independent reflections
Radiation source: fine-focus sealed tube	7397 reflections with *I* > 2σ(*I*)
Graphite monochromator	*R*_int_ = 0.033
φ and ω scans	θ_max_ = 30.0°, θ_min_ = 1.8°
Absorption correction: multi-scan (*SADABS*; Bruker, 2005)	*h* = −16→16
*T*_min_ = 0.723, *T*_max_ = 0.906	*k* = −16→17
34412 measured reflections	*l* = −18→18

### Refinement

**Table:**

Refinement on *F*^2^	Primary atom site location: structure-invariant direct methods
Least-squares matrix: full	Secondary atom site location: difference Fourier map
*R*[*F*^2^ > 2σ(*F*^2^)] = 0.041	Hydrogen site location: inferred from neighbouring sites
*wR*(*F*^2^) = 0.107	H atoms treated by a mixture of independent and constrained refinement
*S* = 1.03	*w* = 1/[σ^2^(*F*_o_^2^) + (0.0498*P*)^2^ + 0.8476*P*] where *P* = (*F*_o_^2^ + 2*F*_c_^2^)/3
9435 reflections	(Δ/σ)_max_ = 0.002
481 parameters	Δρ_max_ = 0.63 e Å^−^^3^
25 restraints	Δρ_min_ = −0.37 e Å^−^^3^

### Special details

**Table:**

Geometry. All e.s.d.'s (except the e.s.d. in the dihedral angle between two l.s. planes) are estimated using the full covariance matrix. The cell e.s.d.'s are taken into account individually in the estimation of e.s.d.'s in distances, angles and torsion angles; correlations between e.s.d.'s in cell parameters are only used when they are defined by crystal symmetry. An approximate (isotropic) treatment of cell e.s.d.'s is used for estimating e.s.d.'s involving l.s. planes.
Refinement. Refinement of *F*^2^ against ALL reflections. The weighted *R*-factor *wR* and goodness of fit *S* are based on *F*^2^, conventional *R*-factors *R* are based on *F*, with *F* set to zero for negative *F*^2^. The threshold expression of *F*^2^ > σ(*F*^2^) is used only for calculating *R*-factors(gt) *etc*. and is not relevant to the choice of reflections for refinement. *R*-factors based on *F*^2^ are statistically about twice as large as those based on *F*, and *R*- factors based on ALL data will be even larger.

### Fractional atomic coordinates and isotropic or equivalent isotropic displacement parameters (Å^2^)

**Table:**

	*x*	*y*	*z*	*U*_iso_*/*U*_eq_	Occ. (<1)
Ni1	0.78451 (2)	0.19687 (2)	0.41425 (2)	0.01968 (7)	
S1	0.70744 (4)	0.35633 (4)	0.42739 (5)	0.02561 (11)	
S2	0.65024 (5)	0.08658 (4)	0.47337 (4)	0.02594 (11)	
O1	0.90909 (12)	0.09464 (11)	0.45887 (12)	0.0248 (3)	
O2	0.87054 (12)	0.23656 (11)	0.29881 (12)	0.0241 (3)	
N1	1.12110 (15)	0.15001 (14)	0.60044 (15)	0.0242 (3)	
N2	0.94274 (14)	0.33207 (13)	0.55479 (14)	0.0201 (3)	
N3	0.95747 (15)	0.44742 (13)	0.59700 (14)	0.0223 (3)	
N4	0.86366 (17)	0.58352 (14)	0.58507 (16)	0.0260 (4)	
N5	0.83284 (18)	0.11914 (17)	0.10848 (16)	0.0309 (4)	
N6	0.66127 (14)	0.05595 (13)	0.25591 (14)	0.0199 (3)	
N7	0.55864 (15)	−0.03271 (14)	0.23367 (14)	0.0223 (3)	
N8	0.44456 (16)	−0.10476 (15)	0.32126 (16)	0.0271 (4)	
C1	1.01396 (18)	0.17003 (16)	0.54342 (17)	0.0220 (4)	
C2	1.22052 (18)	0.26024 (16)	0.68514 (17)	0.0228 (4)	
C3	1.34585 (19)	0.27929 (18)	0.75792 (18)	0.0272 (4)	
H3A	1.3761	0.2145	0.7565	0.033*	
C4	1.42602 (19)	0.39775 (18)	0.83346 (19)	0.0302 (4)	
H4A	1.5128	0.4140	0.8849	0.036*	
C5	1.38212 (19)	0.49286 (18)	0.83545 (18)	0.0286 (4)	
H5A	1.4392	0.5726	0.8883	0.034*	
C6	1.25508 (18)	0.47278 (17)	0.76076 (17)	0.0247 (4)	
H6A	1.2254	0.5378	0.7614	0.030*	
C7	1.17367 (18)	0.35500 (16)	0.68558 (16)	0.0220 (4)	
C8	1.04087 (17)	0.29907 (15)	0.59716 (16)	0.0205 (3)	
C9	0.84681 (17)	0.46578 (16)	0.54011 (17)	0.0217 (4)	
C10	0.78016 (19)	0.64753 (17)	0.56415 (18)	0.0248 (4)	
C11	0.8371 (2)	0.77289 (18)	0.6156 (2)	0.0306 (4)	
H11A	0.9271	0.8096	0.6597	0.037*	
C12	0.7638 (2)	0.8445 (2)	0.6031 (2)	0.0362 (5)	
H12A	0.8039	0.9297	0.6383	0.043*	
C13	0.6330 (2)	0.7927 (2)	0.5401 (2)	0.0353 (5)	
H13A	0.5826	0.8416	0.5318	0.042*	
C14	0.5770 (2)	0.6699 (2)	0.4895 (3)	0.0444 (6)	
H14A	0.4869	0.6340	0.4459	0.053*	
C15	0.6487 (2)	0.5963 (2)	0.5005 (2)	0.0418 (6)	
H15A	0.6076	0.5112	0.4645	0.050*	
C16	0.80929 (18)	0.14680 (17)	0.20257 (17)	0.0246 (4)	
C17	0.7387 (2)	0.00967 (19)	0.01605 (18)	0.0292 (4)	
C18	0.7279 (2)	−0.0482 (2)	−0.0946 (2)	0.0383 (5)	
H18A	0.7892	−0.0172	−0.1171	0.046*	
C19	0.6232 (2)	−0.1538 (2)	−0.1715 (2)	0.0385 (5)	
H19A	0.6121	−0.1951	−0.2488	0.046*	
C20	0.5349 (2)	−0.2000 (2)	−0.13809 (19)	0.0347 (5)	
H20A	0.4647	−0.2725	−0.1927	0.042*	
C21	0.5470 (2)	−0.14246 (18)	−0.02595 (18)	0.0289 (4)	
H21A	0.4864	−0.1750	−0.0032	0.035*	
C22	0.64997 (19)	−0.03614 (17)	0.05159 (17)	0.0248 (4)	
C23	0.69379 (17)	0.04848 (16)	0.17166 (16)	0.0217 (4)	
C24	0.54556 (18)	−0.02168 (16)	0.33181 (17)	0.0236 (4)	
C25	0.35014 (19)	−0.21055 (18)	0.22267 (18)	0.0282 (4)	
C26	0.3149 (2)	−0.2297 (2)	0.10356 (19)	0.0317 (4)	
H26A	0.3559	−0.1699	0.0835	0.038*	
C27	0.2200 (2)	−0.3359 (2)	0.0138 (2)	0.0381 (5)	
H27A	0.1963	−0.3484	−0.0676	0.046*	
C28	0.1592 (2)	−0.4240 (2)	0.0413 (2)	0.0438 (6)	
H28A	0.0950	−0.4971	−0.0207	0.053*	
C29	0.1926 (2)	−0.4047 (2)	0.1598 (2)	0.0456 (6)	
H29A	0.1500	−0.4644	0.1790	0.055*	
C30	0.2880 (2)	−0.2989 (2)	0.2512 (2)	0.0361 (5)	
H30A	0.3109	−0.2865	0.3325	0.043*	
N9	0.8695 (2)	0.7307 (2)	0.0180 (3)	0.0608 (7)	
C33	0.8769 (3)	0.7217 (4)	−0.0834 (4)	0.0751 (11)	
H33A	0.8143	0.6683	−0.1624	0.090*	0.486 (15)
H33B	0.7932	0.6948	−0.1495	0.090*	0.514 (15)
O3	0.9859 (7)	0.7999 (10)	−0.0528 (10)	0.070 (2)	0.486 (15)
C31	0.7511 (15)	0.6693 (17)	0.0101 (17)	0.113 (6)	0.486 (15)
H31A	0.6901	0.6178	−0.0736	0.169*	0.486 (15)
H31B	0.7179	0.7284	0.0404	0.169*	0.486 (15)
H31C	0.7640	0.6197	0.0583	0.169*	0.486 (15)
C32	0.9682 (15)	0.8108 (15)	0.1582 (11)	0.140 (7)	0.486 (15)
H32A	1.0540	0.8423	0.1681	0.210*	0.486 (15)
H32B	0.9671	0.7606	0.2052	0.210*	0.486 (15)
H32C	0.9430	0.8781	0.1861	0.210*	0.486 (15)
O3X	0.9563 (5)	0.7356 (8)	−0.1188 (8)	0.056 (2)	0.514 (15)
C31X	0.7529 (9)	0.6800 (9)	0.0153 (8)	0.040 (2)	0.514 (15)
H31D	0.6858	0.6375	−0.0680	0.061*	0.514 (15)
H31E	0.7319	0.7441	0.0564	0.061*	0.514 (15)
H31F	0.7600	0.6232	0.0558	0.061*	0.514 (15)
C32X	0.9868 (9)	0.7923 (8)	0.1185 (9)	0.070 (3)	0.514 (15)
H32D	1.0535	0.7815	0.0979	0.105*	0.514 (15)
H32E	0.9902	0.7609	0.1808	0.105*	0.514 (15)
H32F	1.0001	0.8782	0.1483	0.105*	0.514 (15)
H1N8	0.442 (2)	−0.092 (2)	0.388 (2)	0.037 (7)*	
H1N1	1.127 (3)	0.086 (3)	0.590 (2)	0.045 (8)*	
H1N5	0.901 (3)	0.162 (3)	0.107 (3)	0.055 (8)*	
H1N4	0.938 (2)	0.627 (2)	0.630 (2)	0.033 (7)*	

### Atomic displacement parameters (Å^2^)

**Table:**

	*U*^11^	*U*^22^	*U*^33^	*U*^12^	*U*^13^	*U*^23^
Ni1	0.01728 (12)	0.01467 (11)	0.02259 (12)	0.00379 (9)	0.00726 (9)	0.00553 (9)
S1	0.0183 (2)	0.0182 (2)	0.0310 (2)	0.00567 (18)	0.00575 (19)	0.00578 (19)
S2	0.0247 (2)	0.0222 (2)	0.0242 (2)	0.00144 (19)	0.0111 (2)	0.00564 (18)
O1	0.0202 (7)	0.0157 (6)	0.0281 (7)	0.0044 (5)	0.0050 (6)	0.0051 (5)
O2	0.0210 (7)	0.0196 (6)	0.0250 (7)	0.0007 (5)	0.0099 (6)	0.0057 (5)
N1	0.0217 (8)	0.0147 (7)	0.0282 (8)	0.0075 (6)	0.0055 (7)	0.0054 (6)
N2	0.0200 (8)	0.0149 (7)	0.0234 (7)	0.0055 (6)	0.0090 (6)	0.0069 (6)
N3	0.0204 (8)	0.0141 (7)	0.0272 (8)	0.0057 (6)	0.0081 (6)	0.0056 (6)
N4	0.0191 (8)	0.0163 (7)	0.0359 (9)	0.0069 (7)	0.0087 (7)	0.0065 (7)
N5	0.0284 (9)	0.0325 (9)	0.0300 (9)	0.0049 (8)	0.0165 (8)	0.0103 (8)
N6	0.0156 (7)	0.0163 (7)	0.0244 (8)	0.0043 (6)	0.0075 (6)	0.0073 (6)
N7	0.0182 (8)	0.0192 (7)	0.0256 (8)	0.0038 (6)	0.0094 (6)	0.0071 (6)
N8	0.0243 (9)	0.0248 (8)	0.0254 (8)	0.0006 (7)	0.0117 (7)	0.0065 (7)
C1	0.0213 (9)	0.0161 (8)	0.0246 (9)	0.0070 (7)	0.0078 (7)	0.0064 (7)
C2	0.0216 (9)	0.0174 (8)	0.0240 (9)	0.0062 (7)	0.0074 (7)	0.0060 (7)
C3	0.0243 (10)	0.0220 (9)	0.0305 (10)	0.0101 (8)	0.0081 (8)	0.0092 (8)
C4	0.0219 (10)	0.0269 (10)	0.0308 (10)	0.0064 (8)	0.0043 (8)	0.0095 (8)
C5	0.0238 (10)	0.0200 (9)	0.0281 (10)	0.0027 (8)	0.0045 (8)	0.0054 (8)
C6	0.0251 (10)	0.0182 (8)	0.0262 (9)	0.0067 (8)	0.0096 (8)	0.0066 (7)
C7	0.0209 (9)	0.0187 (8)	0.0228 (9)	0.0067 (7)	0.0075 (7)	0.0076 (7)
C8	0.0199 (9)	0.0144 (8)	0.0231 (9)	0.0051 (7)	0.0075 (7)	0.0061 (7)
C9	0.0203 (9)	0.0170 (8)	0.0263 (9)	0.0060 (7)	0.0106 (7)	0.0075 (7)
C10	0.0253 (10)	0.0208 (9)	0.0308 (10)	0.0118 (8)	0.0134 (8)	0.0103 (8)
C11	0.0263 (10)	0.0213 (9)	0.0406 (12)	0.0105 (8)	0.0136 (9)	0.0082 (9)
C12	0.0397 (13)	0.0257 (10)	0.0477 (13)	0.0180 (10)	0.0223 (11)	0.0128 (10)
C13	0.0365 (12)	0.0351 (11)	0.0456 (13)	0.0238 (10)	0.0219 (10)	0.0192 (10)
C14	0.0260 (11)	0.0378 (13)	0.0644 (17)	0.0176 (10)	0.0152 (11)	0.0166 (12)
C15	0.0255 (11)	0.0243 (10)	0.0644 (16)	0.0094 (9)	0.0139 (11)	0.0116 (11)
C16	0.0244 (10)	0.0247 (9)	0.0276 (9)	0.0103 (8)	0.0127 (8)	0.0124 (8)
C17	0.0304 (11)	0.0290 (10)	0.0280 (10)	0.0097 (9)	0.0138 (9)	0.0115 (8)
C18	0.0472 (14)	0.0384 (12)	0.0341 (11)	0.0148 (11)	0.0242 (11)	0.0134 (10)
C19	0.0537 (15)	0.0356 (12)	0.0253 (10)	0.0175 (11)	0.0183 (10)	0.0094 (9)
C20	0.0410 (13)	0.0260 (10)	0.0264 (10)	0.0099 (9)	0.0094 (9)	0.0064 (8)
C21	0.0297 (11)	0.0239 (9)	0.0274 (10)	0.0080 (8)	0.0100 (8)	0.0081 (8)
C22	0.0249 (10)	0.0234 (9)	0.0247 (9)	0.0094 (8)	0.0099 (8)	0.0090 (8)
C23	0.0189 (9)	0.0215 (8)	0.0232 (9)	0.0077 (7)	0.0087 (7)	0.0078 (7)
C24	0.0194 (9)	0.0193 (8)	0.0278 (9)	0.0052 (7)	0.0087 (8)	0.0083 (7)
C25	0.0213 (9)	0.0240 (9)	0.0308 (10)	0.0029 (8)	0.0110 (8)	0.0043 (8)
C26	0.0244 (10)	0.0321 (11)	0.0312 (10)	0.0057 (9)	0.0112 (9)	0.0082 (9)
C27	0.0288 (11)	0.0371 (12)	0.0330 (11)	0.0042 (10)	0.0108 (9)	0.0029 (10)
C28	0.0335 (12)	0.0298 (11)	0.0434 (14)	−0.0027 (10)	0.0131 (11)	−0.0028 (10)
C29	0.0407 (14)	0.0292 (11)	0.0513 (15)	−0.0024 (10)	0.0201 (12)	0.0076 (11)
C30	0.0339 (12)	0.0277 (10)	0.0370 (12)	0.0011 (9)	0.0163 (10)	0.0072 (9)
N9	0.0493 (14)	0.0616 (16)	0.0837 (19)	0.0229 (13)	0.0305 (14)	0.0451 (15)
C33	0.064 (2)	0.104 (3)	0.119 (3)	0.049 (2)	0.062 (2)	0.089 (3)
O3	0.058 (3)	0.091 (6)	0.074 (5)	0.012 (4)	0.048 (4)	0.037 (5)
C31	0.069 (10)	0.143 (13)	0.140 (13)	0.023 (9)	0.065 (9)	0.063 (10)
C32	0.130 (10)	0.151 (13)	0.056 (6)	−0.048 (9)	0.043 (6)	0.001 (7)
O3X	0.038 (2)	0.069 (4)	0.064 (4)	0.006 (2)	0.030 (3)	0.033 (4)
C31X	0.049 (5)	0.063 (4)	0.052 (4)	0.042 (4)	0.037 (4)	0.047 (4)
C32X	0.070 (5)	0.054 (4)	0.048 (5)	0.021 (4)	0.003 (4)	0.004 (4)

### Geometric parameters (Å, º)

**Table:**

Ni1—N2	2.0342 (15)	C14—C15	1.391 (3)
Ni1—N6	2.0373 (15)	C14—H14A	0.9500
Ni1—O1	2.1886 (13)	C15—H15A	0.9500
Ni1—O2	2.2441 (13)	C16—C23	1.475 (3)
Ni1—S1	2.3564 (5)	C17—C18	1.381 (3)
Ni1—S2	2.3866 (5)	C17—C22	1.405 (3)
S1—C9	1.7053 (19)	C18—C19	1.390 (3)
S2—C24	1.728 (2)	C18—H18A	0.9500
O1—C1	1.255 (2)	C19—C20	1.382 (3)
O2—C16	1.248 (2)	C19—H19A	0.9500
N1—C1	1.347 (2)	C20—C21	1.392 (3)
N1—C2	1.414 (2)	C20—H20A	0.9500
N1—H1N1	0.79 (3)	C21—C22	1.388 (3)
N2—C8	1.315 (2)	C21—H21A	0.9500
N2—N3	1.328 (2)	C22—C23	1.451 (3)
N3—C9	1.371 (2)	C25—C26	1.386 (3)
N4—C9	1.352 (2)	C25—C30	1.403 (3)
N4—C10	1.412 (2)	C26—C27	1.385 (3)
N4—H1N4	0.81 (3)	C26—H26A	0.9500
N5—C16	1.352 (3)	C27—C28	1.384 (4)
N5—C17	1.414 (3)	C27—H27A	0.9500
N5—H1N5	0.89 (3)	C28—C29	1.383 (4)
N6—C23	1.302 (2)	C28—H28A	0.9500
N6—N7	1.342 (2)	C29—C30	1.390 (3)
N7—C24	1.335 (2)	C29—H29A	0.9500
N8—C24	1.353 (2)	C30—H30A	0.9500
N8—C25	1.418 (3)	N9—C33	1.347 (4)
N8—H1N8	0.85 (3)	N9—C32X	1.381 (9)
C1—C8	1.464 (2)	N9—C31X	1.408 (10)
C2—C3	1.381 (3)	N9—C31	1.429 (16)
C2—C7	1.410 (2)	N9—C32	1.619 (12)
C3—C4	1.393 (3)	C33—O3X	1.229 (6)
C3—H3A	0.9500	C33—O3	1.298 (8)
C4—C5	1.392 (3)	C33—H33A	0.9500
C4—H4A	0.9500	C33—H33B	0.9600
C5—C6	1.400 (3)	C31—H31A	0.9800
C5—H5A	0.9500	C31—H31B	0.9800
C6—C7	1.389 (3)	C31—H31C	0.9800
C6—H6A	0.9500	C32—H32A	0.9800
C7—C8	1.445 (3)	C32—H32B	0.9800
C10—C15	1.386 (3)	C32—H32C	0.9800
C10—C11	1.396 (3)	C31X—H31D	0.9800
C11—C12	1.386 (3)	C31X—H31E	0.9800
C11—H11A	0.9500	C31X—H31F	0.9800
C12—C13	1.379 (3)	C32X—H32D	0.9800
C12—H12A	0.9500	C32X—H32E	0.9800
C13—C14	1.367 (3)	C32X—H32F	0.9800
C13—H13A	0.9500		
			
N2—Ni1—N6	163.87 (6)	C10—C15—H15A	120.1
N2—Ni1—O1	80.64 (5)	C14—C15—H15A	120.1
N6—Ni1—O1	89.97 (5)	O2—C16—N5	128.61 (19)
N2—Ni1—O2	86.61 (5)	O2—C16—C23	124.53 (17)
N6—Ni1—O2	79.65 (5)	N5—C16—C23	106.86 (17)
O1—Ni1—O2	86.10 (5)	C18—C17—C22	122.1 (2)
N2—Ni1—S1	80.27 (4)	C18—C17—N5	128.4 (2)
N6—Ni1—S1	109.33 (4)	C22—C17—N5	109.45 (18)
O1—Ni1—S1	160.66 (4)	C17—C18—C19	117.1 (2)
O2—Ni1—S1	95.85 (4)	C17—C18—H18A	121.4
N2—Ni1—S2	113.35 (5)	C19—C18—H18A	121.4
N6—Ni1—S2	79.44 (4)	C20—C19—C18	121.4 (2)
O1—Ni1—S2	89.75 (4)	C20—C19—H19A	119.3
O2—Ni1—S2	158.67 (4)	C18—C19—H19A	119.3
S1—Ni1—S2	94.949 (19)	C19—C20—C21	121.3 (2)
C9—S1—Ni1	96.51 (6)	C19—C20—H20A	119.3
C24—S2—Ni1	95.92 (7)	C21—C20—H20A	119.3
C1—O1—Ni1	105.85 (11)	C22—C21—C20	118.1 (2)
C16—O2—Ni1	104.98 (12)	C22—C21—H21A	121.0
C1—N1—C2	110.03 (15)	C20—C21—H21A	121.0
C1—N1—H1N1	125 (2)	C21—C22—C17	119.86 (19)
C2—N1—H1N1	125 (2)	C21—C22—C23	134.39 (19)
C8—N2—N3	119.43 (15)	C17—C22—C23	105.75 (17)
C8—N2—Ni1	113.80 (12)	N6—C23—C22	137.10 (18)
N3—N2—Ni1	126.43 (12)	N6—C23—C16	115.45 (16)
N2—N3—C9	111.47 (15)	C22—C23—C16	107.37 (16)
C9—N4—C10	133.07 (18)	N7—C24—N8	117.60 (17)
C9—N4—H1N4	113.1 (17)	N7—C24—S2	125.57 (15)
C10—N4—H1N4	113.6 (17)	N8—C24—S2	116.77 (15)
C16—N5—C17	110.54 (17)	C26—C25—C30	119.45 (19)
C16—N5—H1N5	124.5 (19)	C26—C25—N8	124.72 (19)
C17—N5—H1N5	124.9 (19)	C30—C25—N8	115.81 (19)
C23—N6—N7	117.97 (16)	C27—C26—C25	120.1 (2)
C23—N6—Ni1	114.66 (12)	C27—C26—H26A	120.0
N7—N6—Ni1	127.13 (12)	C25—C26—H26A	120.0
C24—N7—N6	111.61 (15)	C26—C27—C28	120.8 (2)
C24—N8—C25	130.78 (18)	C26—C27—H27A	119.6
C24—N8—H1N8	112.5 (17)	C28—C27—H27A	119.6
C25—N8—H1N8	116.5 (17)	C29—C28—C27	119.4 (2)
O1—C1—N1	127.81 (17)	C29—C28—H28A	120.3
O1—C1—C8	124.69 (16)	C27—C28—H28A	120.3
N1—C1—C8	107.50 (16)	C28—C29—C30	120.6 (2)
C3—C2—C7	122.46 (17)	C28—C29—H29A	119.7
C3—C2—N1	128.16 (17)	C30—C29—H29A	119.7
C7—C2—N1	109.37 (16)	C29—C30—C25	119.6 (2)
C2—C3—C4	117.01 (18)	C29—C30—H30A	120.2
C2—C3—H3A	121.5	C25—C30—H30A	120.2
C4—C3—H3A	121.5	C33—N9—C32X	111.8 (6)
C5—C4—C3	121.63 (19)	C33—N9—C31X	121.5 (4)
C5—C4—H4A	119.2	C32X—N9—C31X	126.7 (6)
C3—C4—H4A	119.2	C33—N9—C31	119.3 (8)
C4—C5—C6	120.96 (18)	C32X—N9—C31	128.6 (10)
C4—C5—H5A	119.5	C33—N9—C32	132.9 (6)
C6—C5—H5A	119.5	C31X—N9—C32	104.9 (7)
C7—C6—C5	118.11 (17)	C31—N9—C32	107.6 (9)
C7—C6—H6A	120.9	O3X—C33—N9	140.3 (5)
C5—C6—H6A	120.9	O3—C33—N9	107.0 (6)
C6—C7—C2	119.83 (17)	O3X—C33—H33A	89.3
C6—C7—C8	134.55 (17)	O3—C33—H33A	126.5
C2—C7—C8	105.60 (15)	N9—C33—H33A	126.5
N2—C8—C7	137.86 (17)	O3X—C33—H33B	109.8
N2—C8—C1	114.74 (16)	O3—C33—H33B	133.6
C7—C8—C1	107.39 (15)	N9—C33—H33B	109.9
N4—C9—N3	110.48 (16)	N9—C31—H31A	109.5
N4—C9—S1	124.46 (14)	N9—C31—H31B	109.5
N3—C9—S1	125.06 (14)	N9—C31—H31C	109.5
C15—C10—C11	118.47 (18)	N9—C32—H32A	109.5
C15—C10—N4	125.52 (18)	N9—C32—H32B	109.5
C11—C10—N4	115.98 (18)	N9—C32—H32C	109.5
C12—C11—C10	120.7 (2)	N9—C31X—H31D	109.5
C12—C11—H11A	119.6	N9—C31X—H31E	109.5
C10—C11—H11A	119.6	H31D—C31X—H31E	109.5
C13—C12—C11	120.4 (2)	N9—C31X—H31F	109.5
C13—C12—H12A	119.8	H31D—C31X—H31F	109.5
C11—C12—H12A	119.8	H31E—C31X—H31F	109.5
C14—C13—C12	119.0 (2)	N9—C32X—H32D	109.5
C14—C13—H13A	120.5	N9—C32X—H32E	109.5
C12—C13—H13A	120.5	H32D—C32X—H32E	109.5
C13—C14—C15	121.6 (2)	N9—C32X—H32F	109.5
C13—C14—H14A	119.2	H32D—C32X—H32F	109.5
C15—C14—H14A	119.2	H32E—C32X—H32F	109.5
C10—C15—C14	119.8 (2)		
			
N2—Ni1—S1—C9	3.18 (8)	O1—C1—C8—C7	−176.31 (18)
N6—Ni1—S1—C9	−163.42 (8)	N1—C1—C8—C7	3.1 (2)
O1—Ni1—S1—C9	12.56 (14)	C10—N4—C9—N3	−175.2 (2)
O2—Ni1—S1—C9	−82.36 (7)	C10—N4—C9—S1	5.4 (3)
S2—Ni1—S1—C9	116.05 (7)	N2—N3—C9—N4	179.52 (16)
N2—Ni1—S2—C24	−173.88 (8)	N2—N3—C9—S1	−1.1 (2)
N6—Ni1—S2—C24	−4.18 (7)	Ni1—S1—C9—N4	177.06 (16)
O1—Ni1—S2—C24	−94.19 (7)	Ni1—S1—C9—N3	−2.25 (17)
O2—Ni1—S2—C24	−15.62 (13)	C9—N4—C10—C15	11.0 (4)
S1—Ni1—S2—C24	104.59 (6)	C9—N4—C10—C11	−170.9 (2)
N2—Ni1—O1—C1	−2.40 (12)	C15—C10—C11—C12	−0.1 (3)
N6—Ni1—O1—C1	164.43 (13)	N4—C10—C11—C12	−178.4 (2)
O2—Ni1—O1—C1	84.80 (12)	C10—C11—C12—C13	0.4 (4)
S1—Ni1—O1—C1	−11.8 (2)	C11—C12—C13—C14	−0.4 (4)
S2—Ni1—O1—C1	−116.13 (12)	C12—C13—C14—C15	0.1 (4)
N2—Ni1—O2—C16	164.17 (12)	C11—C10—C15—C14	−0.1 (4)
N6—Ni1—O2—C16	−7.35 (12)	N4—C10—C15—C14	178.0 (2)
O1—Ni1—O2—C16	83.33 (12)	C13—C14—C15—C10	0.1 (4)
S1—Ni1—O2—C16	−115.98 (11)	Ni1—O2—C16—N5	−171.38 (18)
S2—Ni1—O2—C16	4.09 (19)	Ni1—O2—C16—C23	8.4 (2)
N6—Ni1—N2—C8	−50.5 (3)	C17—N5—C16—O2	−179.06 (19)
O1—Ni1—N2—C8	4.60 (13)	C17—N5—C16—C23	1.1 (2)
O2—Ni1—N2—C8	−82.01 (13)	C16—N5—C17—C18	179.1 (2)
S1—Ni1—N2—C8	−178.54 (13)	C16—N5—C17—C22	−0.1 (2)
S2—Ni1—N2—C8	90.24 (13)	C22—C17—C18—C19	1.2 (3)
N6—Ni1—N2—N3	122.6 (2)	N5—C17—C18—C19	−177.9 (2)
O1—Ni1—N2—N3	177.76 (16)	C17—C18—C19—C20	−1.0 (3)
O2—Ni1—N2—N3	91.15 (15)	C18—C19—C20—C21	0.2 (4)
S1—Ni1—N2—N3	−5.38 (14)	C19—C20—C21—C22	0.6 (3)
S2—Ni1—N2—N3	−96.60 (14)	C20—C21—C22—C17	−0.5 (3)
C8—N2—N3—C9	178.00 (16)	C20—C21—C22—C23	179.3 (2)
Ni1—N2—N3—C9	5.2 (2)	C18—C17—C22—C21	−0.4 (3)
N2—Ni1—N6—C23	−26.1 (3)	N5—C17—C22—C21	178.82 (18)
O1—Ni1—N6—C23	−80.17 (13)	C18—C17—C22—C23	179.8 (2)
O2—Ni1—N6—C23	5.88 (12)	N5—C17—C22—C23	−1.0 (2)
S1—Ni1—N6—C23	98.50 (13)	N7—N6—C23—C22	−2.1 (3)
S2—Ni1—N6—C23	−169.91 (13)	Ni1—N6—C23—C22	172.66 (19)
N2—Ni1—N6—N7	148.11 (19)	N7—N6—C23—C16	−178.39 (15)
O1—Ni1—N6—N7	94.07 (14)	Ni1—N6—C23—C16	−3.6 (2)
O2—Ni1—N6—N7	−179.88 (15)	C21—C22—C23—N6	5.4 (4)
S1—Ni1—N6—N7	−87.26 (14)	C17—C22—C23—N6	−174.8 (2)
S2—Ni1—N6—N7	4.33 (13)	C21—C22—C23—C16	−178.1 (2)
C23—N6—N7—C24	172.15 (16)	C17—C22—C23—C16	1.6 (2)
Ni1—N6—N7—C24	−1.9 (2)	O2—C16—C23—N6	−4.2 (3)
Ni1—O1—C1—N1	−179.24 (18)	N5—C16—C23—N6	175.61 (16)
Ni1—O1—C1—C8	0.1 (2)	O2—C16—C23—C22	178.46 (18)
C2—N1—C1—O1	176.12 (19)	N5—C16—C23—C22	−1.7 (2)
C2—N1—C1—C8	−3.3 (2)	N6—N7—C24—N8	179.18 (16)
C1—N1—C2—C3	−177.1 (2)	N6—N7—C24—S2	−3.5 (2)
C1—N1—C2—C7	2.3 (2)	C25—N8—C24—N7	4.8 (3)
C7—C2—C3—C4	0.1 (3)	C25—N8—C24—S2	−172.74 (17)
N1—C2—C3—C4	179.3 (2)	Ni1—S2—C24—N7	5.77 (17)
C2—C3—C4—C5	−0.2 (3)	Ni1—S2—C24—N8	−176.93 (14)
C3—C4—C5—C6	−0.3 (3)	C24—N8—C25—C26	−24.4 (3)
C4—C5—C6—C7	0.8 (3)	C24—N8—C25—C30	157.2 (2)
C5—C6—C7—C2	−0.8 (3)	C30—C25—C26—C27	−0.7 (3)
C5—C6—C7—C8	−179.1 (2)	N8—C25—C26—C27	−179.1 (2)
C3—C2—C7—C6	0.4 (3)	C25—C26—C27—C28	0.0 (4)
N1—C2—C7—C6	−178.94 (17)	C26—C27—C28—C29	0.9 (4)
C3—C2—C7—C8	179.16 (18)	C27—C28—C29—C30	−1.1 (4)
N1—C2—C7—C8	−0.2 (2)	C28—C29—C30—C25	0.5 (4)
N3—N2—C8—C7	0.8 (3)	C26—C25—C30—C29	0.5 (3)
Ni1—N2—C8—C7	174.5 (2)	N8—C25—C30—C29	179.0 (2)
N3—N2—C8—C1	−179.47 (16)	C32X—N9—C33—O3X	13.7 (9)
Ni1—N2—C8—C1	−5.8 (2)	C31X—N9—C33—O3X	−165.7 (8)
C6—C7—C8—N2	−3.5 (4)	C31—N9—C33—O3X	−160.5 (10)
C2—C7—C8—N2	178.0 (2)	C32—N9—C33—O3X	26.4 (12)
C6—C7—C8—C1	176.7 (2)	C32X—N9—C33—O3	−16.0 (6)
C2—C7—C8—C1	−1.7 (2)	C31X—N9—C33—O3	164.6 (5)
O1—C1—C8—N2	3.9 (3)	C31—N9—C33—O3	169.7 (9)
N1—C1—C8—N2	−176.67 (16)	C32—N9—C33—O3	−3.3 (10)

### Hydrogen-bond geometry (Å, º)

*Cg*5, *Cg*7, *Cg*9 and *Cg*10 are the centroids of the N1/C1/C8/C7/C2, C2–C7, C17–C22 and C25–C30 rings, respectively.

**Table:**

*D*—H···*A*	*D*—H	H···*A*	*D*···*A*	*D*—H···*A*
N8—H1*N*8···S2^i^	0.86 (2)	2.48 (3)	3.301 (2)	159 (2)
N1—H1*N*1···O1^ii^	0.79 (4)	2.04 (4)	2.811 (2)	165 (4)
N5—H1*N*5···O3*X*^iii^	0.89 (4)	1.84 (4)	2.729 (9)	173 (3)
N4—H1*N*4···O2^iv^	0.82 (3)	2.22 (3)	3.006 (3)	161 (2)
C3—H3*A*···N7^ii^	0.95	2.56	3.500 (3)	173
C11—H11*A*···O2^iv^	0.95	2.55	3.349 (3)	142
C15—H15*A*···S1	0.95	2.53	3.194 (3)	127
C20—H20*A*···S1^v^	0.95	2.71	3.443 (2)	135
C26—H26*A*···N7	0.95	2.35	2.900 (3)	116
C30—H30*A*···S2^i^	0.95	2.84	3.551 (2)	132
C32*X*—H32*D*···O3*X*	0.98	2.46	2.859 (14)	104
C31*X*—H31*E*···*Cg*9^vi^	0.98	2.97	3.567 (11)	121
C31*X*—H31*F*···*Cg*7^iv^	0.98	2.79	3.460 (10)	126
C32*X*—H32*D*···*Cg*10^vii^	0.98	2.91	3.769 (12)	147
C31—H31*B*···*Cg*9^vi^	0.98	2.98	3.66 (2)	128
C31—H31*C*···*Cg*7^iv^	0.98	2.78	3.47 (2)	127
C32—H32*B*···*Cg*5^iv^	0.98	2.82	3.577 (16)	135

Symmetry codes: (i) −*x*+1, −*y*, −*z*+1; (ii) −*x*+2, −*y*, −*z*+1; (iii) −*x*+2, −*y*+1, −*z*; (iv) −*x*+2, −*y*+1, −*z*+1; (v) −*x*+1, −*y*, −*z*; (vi) *x*, *y*+1, *z*; (vii) *x*+1, *y*+1, *z*.
